# Case Report: Tislelizumab-induced delayed-onset diabetic ketoacidosis and hypothyroidism in esophageal adenocarcinoma

**DOI:** 10.3389/fonc.2025.1642893

**Published:** 2025-08-28

**Authors:** Zhaoyang Li, Kangning Han, Li Li, Xiaochun Han, Feng Zhang, Liangqing Guo

**Affiliations:** ^1^ The First Clinical Medical College, Shandong University of Traditional Chinese Medicine, Jinan, China; ^2^ Department of Endocrinology, Affiliated Hospital of Shandong University of Traditional Chinese Medicine, Jinan, China; ^3^ College of Health, Shandong University of Traditional Chinese Medicine, Jinan, China; ^4^ Department of Ultrasound, Affiliated Hospital of Shandong University of Traditional Chinese Medicine, Jinan, China

**Keywords:** tislelizumab, esophageal adenocarcinoma, diabetic ketoacidosis, immune checkpoint inhibitors, case report

## Abstract

Immune checkpoint inhibitor-related diabetes ketoacidosis (ICI-DKA) and hypothyroidism are rare but serious adverse events of Anti-programmed cell death-1 (anti-PD-1) therapy, previously unreported in patients with EAC receiving tislelizumab. A 53-year-old man with well-controlled type 2 diabetes and human epidermal growth factor receptor 2 (HER2)-positive metastatic EAC completed 8 cycles of tislelizumab combined with trastuzumab and capecitabine-oxaliplatin (XELOX) chemotherapy, achieving a partial response. Eleven months after the last tislelizumab dose, he presented with fatigue and was diagnosed with DKA and hypothyroidism. Laboratory testing revealed severe insulin deficiency and negative diabetes-related autoantibodies. This was the first reported case of tislelizumab-induced concurrent DKA and hypothyroidism in an EAC patient. This case demonstrates the potential for severe, delayed-onset endocrine toxicity with tislelizumab, even in patients with pre-existing diabetes. It underscores the necessity for extended monitoring of glucose, C-peptide, and thyroid function beyond active therapy in programmed cell death protein 1 (PD-1) inhibitor recipients to mitigate life-threatening complications like ICI-DKA. Given the expanding global use of tislelizumab for EAC and the life-threatening nature of ICI-DKA, this case provides critical safety evidence necessitating urgent protocol revisions for long-term endocrine monitoring.

## 1 Introduction

Esophageal cancer is the sixth leading cause of global cancer-related deaths, with squamous cell carcinoma (SCC) and adenocarcinoma (AC) as the predominant subtypes. In high-income countries, the incidence of EAC has surged, now representing approximately two-thirds of cases (1). Prior to ICI development, HER2 was a key target in EAC (2). These targeted drugs, including monoclonal antibodies, tyrosine kinase inhibitors and antibody-drug conjugates (ADCs) combined with platinum-based chemotherapy, are the traditional treatment methods for HER2-positive EAC.

Neoadjuvant immune checkpoint blockade (ICB) has been widely evaluated in various cancers (3). Consequently, combining ICIs with trastuzumab and platinum-based chemotherapy has emerged as first-line therapy for HER2-positive EAC (4). This regimen significantly improves both overall efficacy and survival (OS), leveraging the unique advantages and potential for long-term disease control with immunotherapy, particularly in subgroups like those with high PD-L1 expression (5). Anti-PD-1 therapy is established for metastatic and adjuvant EAC.

Tislelizumab, an anti-PD-1 antibody independently developed in China, is now integral to treating most resectable EACs due to its survival benefit over surgery alone when combined with chemotherapy (6). However, tislelizumab is associated with adverse events (AEs), including fatigue, rash, hypothyroidism, and transaminitis. ICI-DKA, occurring in 1-2% of cases, is a rare but life-threatening endocrine AE (7). Jeroen et al. reported DKA and autoimmune thyroiditis in a lung cancer patient 8 weeks post-pembrolizumab (7). Reports of tislelizumab-induced ICI-related type 1 diabetes mellitus (ICI-T1DM)/ICI-DKA are limited. A case described by Zhu et al. in small cell lung cancer after 108 days of tislelizumab (8). Xian et al. reported an incident of DKA following tislelizumab treatment in patients with nasopharyngeal carcinoma (9).

Notably, tislelizumab-induced DKA and hypothyroidism have not been previously reported in an EAC patient. We present a case of DKA and hypothyroidism occurring in an EAC patient treated with tislelizumab, trastuzumab, and platinum-based chemotherapy. This report underscores the vital importance of vigilant blood glucose and endocrine hormone monitoring, thorough AE documentation, and heightened clinician awareness of tislelizumab’s adverse effects. These measures are crucial to prevent fatal endocrine-related AEs during tislelizumab therapy for EAC.

## 2 Case description

**Figure 1:**
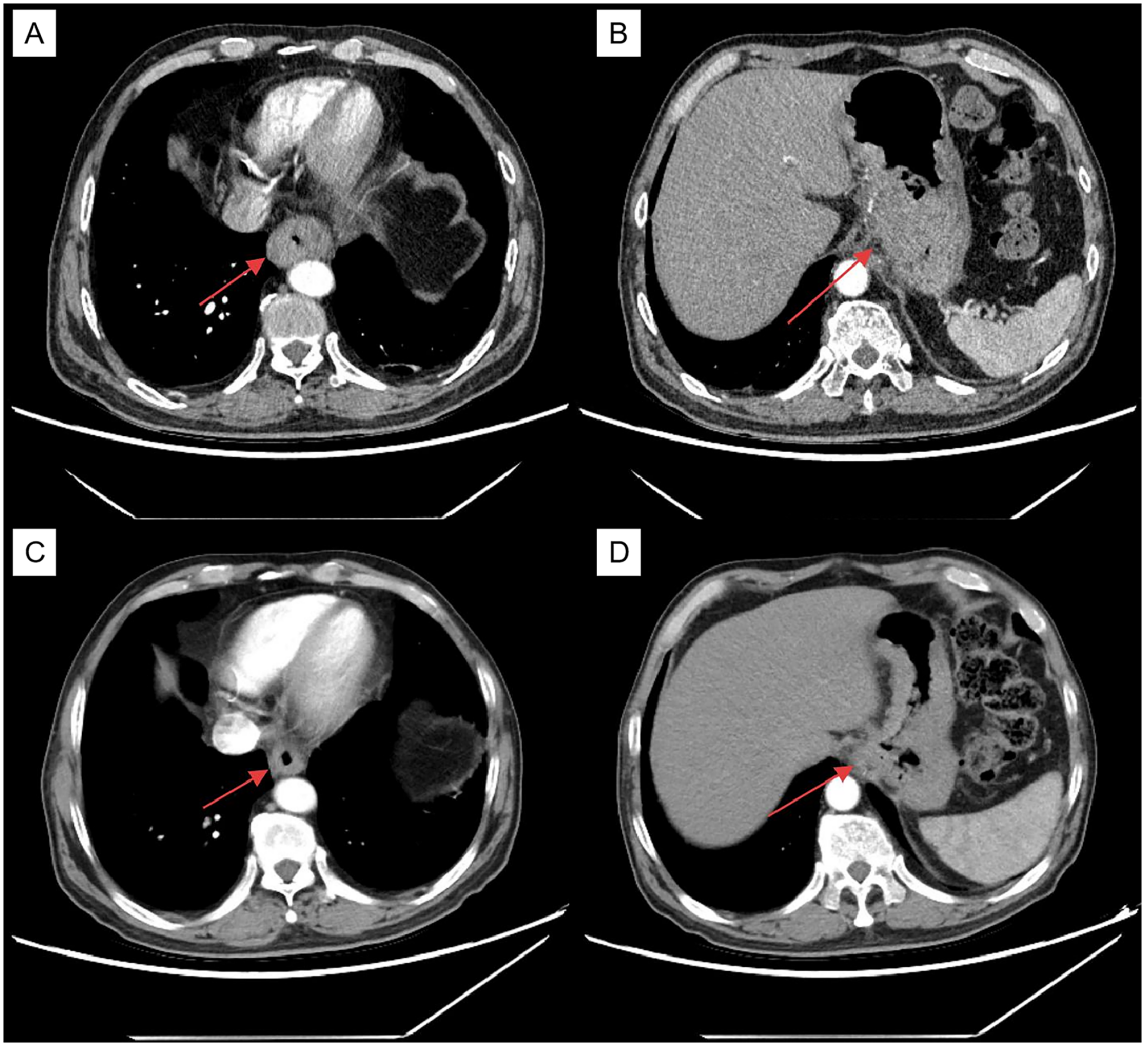
The red arrows indicate: **(A)** Masses in the middle and lower segments of the esophagus before tislelizumab plus XELOX treatment. **(B)** Tislelizumab combined with XELOX for the treatment of anterior gastric masses. **(C)** Masses in the middle and lower segments of the esophagus after four cycles of tislelizumab plus XELOX treatment. **(D)** Gastric masses after four cycles of treatment with tislelizumab plus XELOX.

A 53-year-old male visited the Department of Thoracic Surgery at the Affiliated Hospital of Shandong University of Traditional Chinese Medicine on August 24, 2023, due to a sensation of food obstruction for over two months. The patient had a history of diabetes for 2 years. He took metformin and glimepiride orally with suboptimal glycemic control. The fasting plasma glucose (FPG) measured upon admission was 6.41mmol/L (115.38mg/dL), FT3 was 5.25pmol/L, FT4 was 17.4pmol/L, TSH was 1.31mIU/L. The patient’s HbA1c measured before initiating tislelizumab therapy was 7.1%. Computed tomography (CT) revealed malignant tumors in the lower segment of the esophagus and stomach (**Figures 1A, B**), along with multiple lymph node metastases in the mediastinum, omental sac, hilar, portal space, retroperitoneum, and posterior splenic artery. The pathological results of the patient’s gastroscopy outside the hospital indicated: adenocarcinoma biopsy in the lower segment of the esophagus and moderately to poorly differentiated adenocarcinoma biopsy in the small curvature of the gastric body.

**Figure 2:**
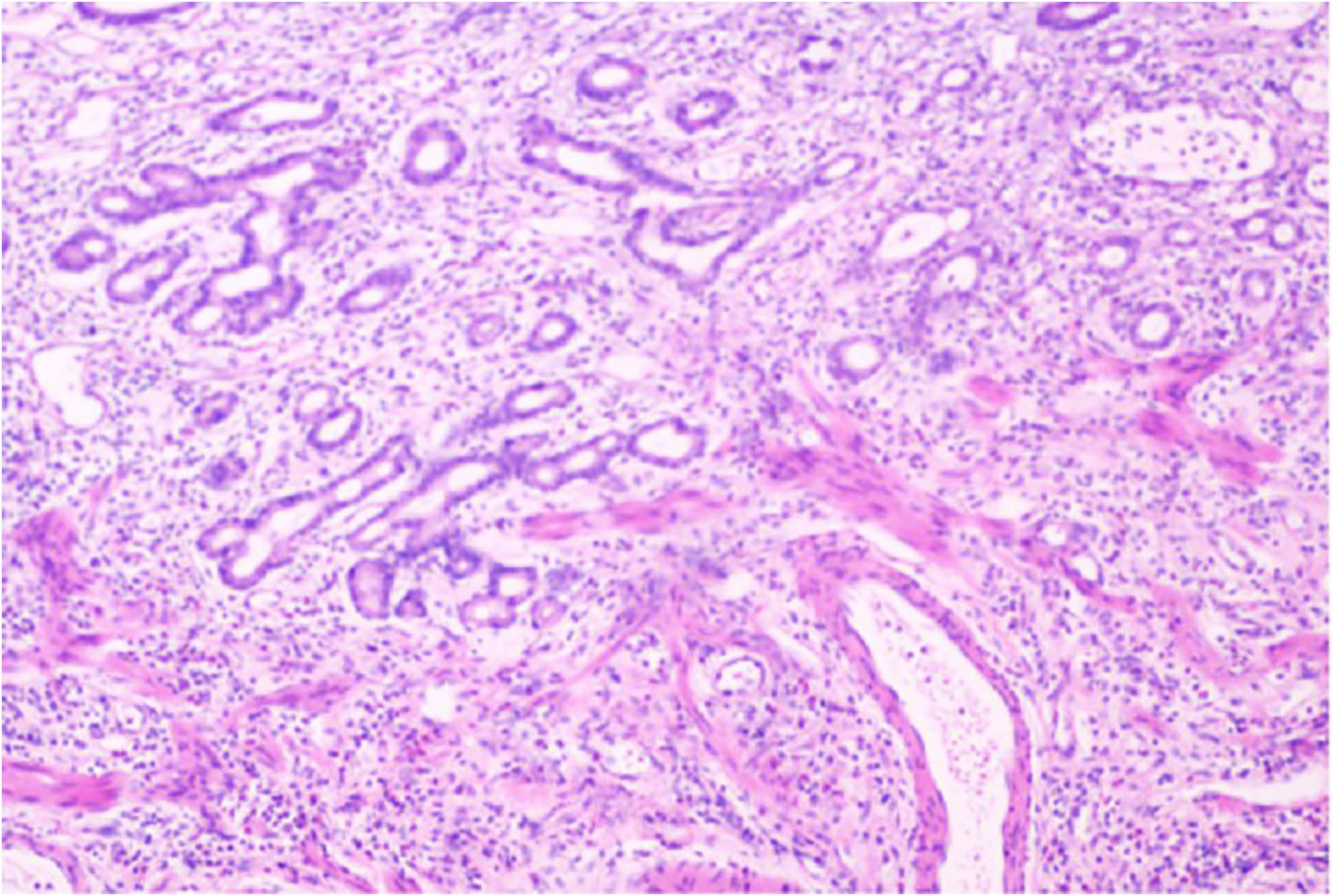
Pathological images of the esophagogastric junction tissues collected after four cycles of tislelizumab plus XELOX treatment and esophageal cancer resection and anastomosis with instruments under the gastroesophageal arch.

On August 27, 2023, the thoracic surgeon treated the patient with tislelizumab combined with XELOX, and the FPG was measured the next day: 6.55mmol/L (118mg/dL). On September 18, 2023, during the second treatment, the genetic test indicated her2 positive. Trastuzumab was added, and the FPG measured at that time was 7.16mmol/L (128.88mg/dL). The FPG at the third treatment on October 11, 2023 was 6.77mmol/L (121.86mg/dL). The FPG at the fourth treatment on November 7, 2023 was 6.65mmol/L (119.7mg/dL). After four treatments, the patient met the surgical indications (**Figures 1C, D**) and underwent esophageal cancer resection and gastroesophageal instrument anastomosis under the arch on December 27, 2023. Pathological examination of the intraoperatively resected specimen revealed a moderately to well-differentiated adenocarcinoma confined to the mucosa. Lymph node dissection yielded 12 lymph nodes, with metastatic carcinoma demonstrating significant treatment response (Grade IV per Hagi criteria) identified in 7 nodes (Pathology No. E202312536). The pathological stage was classified as ypT1aN3M0. The pathology is shown in **Figure 2**, and the detected FPG was 6.68mmol/L (120.24mg/dL). The fifth treatment was received on February 28, 2024, with a FPG of 6.22mmol/L (111.96mg/dL). On March 30, 2024, during the sixth treatment, the FPG was 6.08mmol/L (109.44mg/dL). The seventh treatment was received on May 2, 2024, with a FPG of 6.33mmol/L (113.94mg/dL). The eighth treatment was received on June 5, 2024, with a FPG of 6.17mmol/L (111.06mg/dL).

After eight cycles of tislelizumab treatment, on May 6, 2025, the patient was admitted to the thoracic surgery department of the Affiliated Hospital of Shandong University of Traditional Chinese Medicine due to self-perceived fatigue in the past two months. The laboratory results are shown in (**Table 1**). Blood tests showed that FPG: 13.56mmol/L (244.08mg/dL), β-hydroxybutyric acid: 3.81mmol/L, total carbon dioxide: 10.5mmol/L, anion gap:28mmol/L, bicarbonate concentration: 10.3mmol/L. Urine analysis showed glucose 4+ and ketone body 3+. Metformin and glimepiride were immediately discontinued, and fluid resuscitation combined with insulin therapy was initiated. The patient was subsequently transferred to the Endocrinology Department on 7 May 2025 for further management. The following investigations were conducted: BMI was measured as follows: 23.87kg/m2, fasting C-peptide 0.06ng/ml, fasting insulin < 0.4uU/ml, and Blood ketone body 0.3mmol/L. The autoantibody profile of diabetes is negative. Endocrinologists also found that the patient’s free triiodothyronine was decreased to 2.26pmol/L, thyroid stimulating hormone was increased to6.87μIU/mL, and free thyroxine was 13.5pmol/L, anti-thyroglobulin antibody was 12.2IU/mL, and anti-thyroid peroxidase antibody was12.5IU/mL. Levothyroxine sodium 25 μ5 was administered orally once daily to the patient.

**Table 1:** Laboratory test result.

Investigation	Result	Reference Range	Comment
Fasting blood glucose, mmol/L	13.56	3.9-6.1	High
β-hydroxybutyrate, mmol/L	3.81	0.02-0.27	High
Total carbon dioxide, mmol/L	10.5	22-29	Critically Low
Anion gap, mmol/L	28	8-16	High
Urine glucose	++++	–	High
Urine ketones	+++	–	High
Insulin-fasting, μIU/mL	<0.4	2.6-24.9	Low
C-peptide-fasting, ng/mL	0.06	1.1-4.4	Low
Anti-insulin autoantibodies, COI	0.88	0-1	Normal
Anti-islet cell antibodies, COI	0.08	0-1	Normal
Anti-glutamic acid decarboxylase antibodies, IU/mL	1.05	<10	Normal
Anti-tyrosine phosphatase antibodies, IU/mL	<0.7	<10	Normal
Free triiodothyronine, pmol/L	2.26	3.1-6.8	Low
Free thyroxine, pmol/L	13.5	12.8-21.3	Normal
Thyroid-stimulating hormone, μIU/mL	6.87	0.27-4.2	High
Anti-thyroglobulin antibodies, IU/mL	12.2	0-115	Normal
Anti-thyroid peroxidase antibodies, IU/mL	12.5	0-34	Normal

"+++" indicates a high level (or large amount) of the substance.

"++++" indicates a very high level (or very large amount) of the substance.

**Figure 3:**
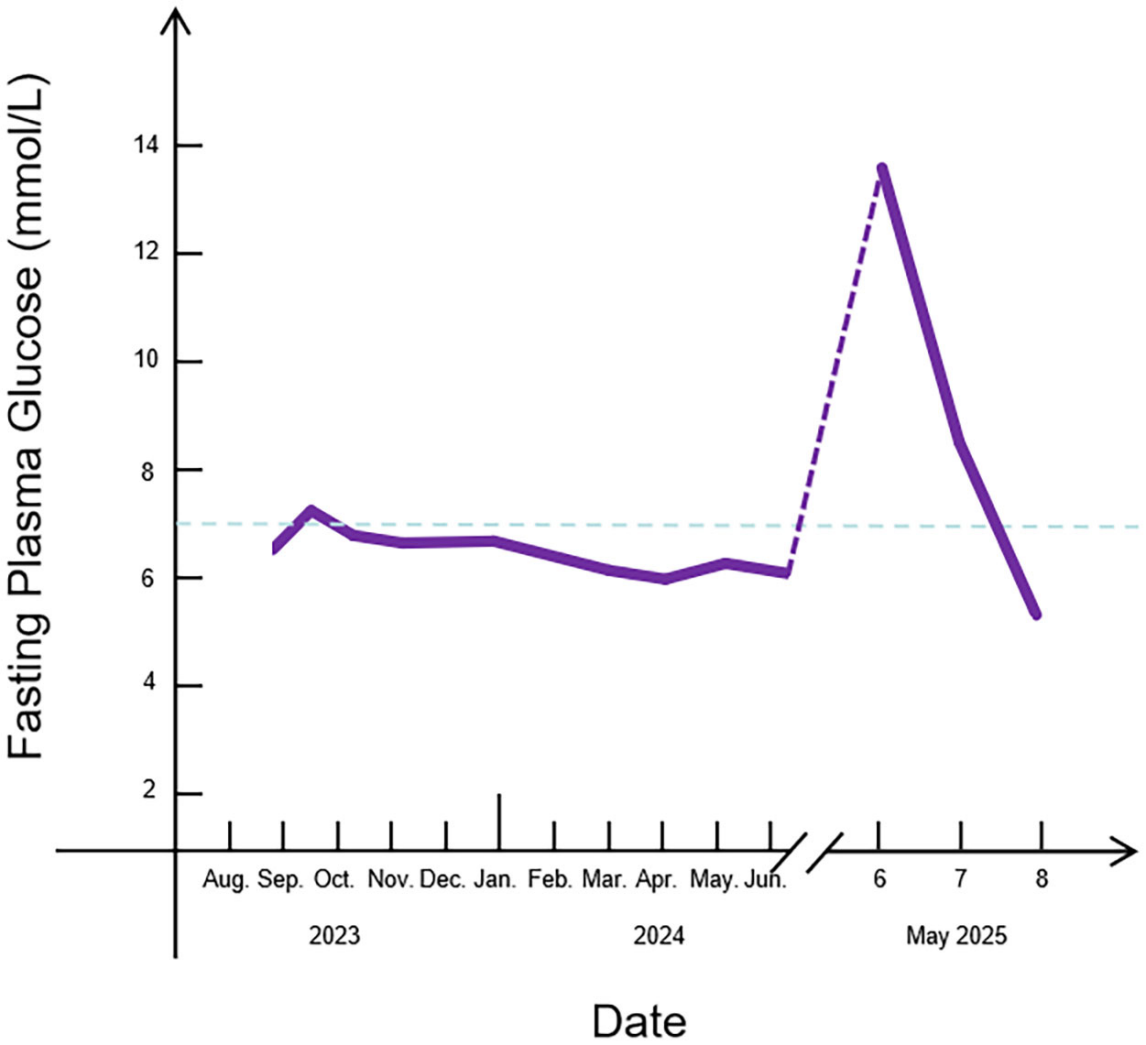
FPG variation trending, 1mmol/L = 18mg/dL.

After two days of fluid replacement and insulin therapy, the patient’s fasting blood glucose dropped to 5.3mmol/L (95.4mg/dL) (**Figure 3**) and stable blood glucose control was achieved. The urine ketone body was negative. On May 9th, the fluid replacement was stopped and 16U of insulin degludec at bedtime was used instead. After subcutaneous injection of insulin aspart 7U before breakfast, 7U before lunch, and 6U before dinner. The patient was advised for hospital discharge and instructed to undergo regular follow-up thyroid function tests after discharge.

## 3 Method and result

We systematically queried PubMed using the following search strategy: (“Tislelizumab Injection”[Title/Abstract] OR “Tislelizumab”[Title/Abstract]) AND (“Diabetic ketoacidosis”[Title/Abstract] OR “DKA”[Title/Abstract] OR “ICI-DKA”[Title/Abstract] OR “type 1 Diabetic ketoacidosis”[Title/Abstract]). This search identified two qualifying case reports (8, 9) involving two distinct patients. Key parameters from these publications—including authors, publication year, patient demographics (age/sex), malignancy type, diabetes history, DKA onset timing, blood glucose levels, and HbA1c percentages—were extracted and comparatively summarized in **Table 2**.

**Table 2:** DKA caused by tislelizumab treatment.

Author	Year	Age at diagnosis	Sex	Type of tumor	History of diabetes	Onset time of DKA	Blood glucose, mmol/l	HbA1c%
Xian, et al (9)	2025	41	Female	nasopharyngeal carcinoma	(–)	13 cycles (21 days per cycle)	33.2	10.2
Zhu, et al (8)	2025	71	Male	extensive-stage small cell lung cancer	(–)	107 days	91.26	10.8

## 4 Discussion

Our findings bridge a critical knowledge gap by reporting the first case of concurrent tislelizumab-induced DKA and hypothyroidism in HER2-positive EAC, particularly notable for its unprecedented 11-month delayed onset after therapy cessation—a phenomenon previously undocumented in this malignancy. The patient had a 2-year history of diabetes mellitus and normal thyroid function at baseline. Blood glucose was controlled within 6.08-7.16 mmol/L during prior cancer treatments. Following 8 treatment cycles over 9 months, the patient gradually developed fatigue, with FPG progressively rising to 13 mmol/L. Oral hypoglycemic agents became ineffective, ultimately leading to severe pancreatic islet failure, DKA, and hypothyroidism. Given that esophagectomy and gastroesophageal anastomosis typically lower rather than elevate blood glucose (10), and considering the patient maintained a diabetic diet and consistent glucose monitoring, these findings exhibit a positive temporal association with tislelizumab administration. This represents a delayed immune-related adverse event (irAE).

ICIs, monoclonal antibodies widely used in cancer therapy, primarily target two key signaling pathways involved in T-cell activation and apoptosis. Cytotoxic T-lymphocyte-associated protein 4 (CTLA-4), programmed cell death protein 1 (PD-1), and programmed death-ligand 1 (PD-L1) are critical regulators of immune tolerance modulated by ICIs. Anti-PD-(L)1 therapy is almost exclusively associated with the development of ICI-T1DM (11). Tislelizumab, a novel humanized anti-PD-1 monoclonal antibody independently developed in China, enhances T-cell-mediated antitumor immune responses by blocking the interaction between PD-1 and its ligands PD-L1 and PD-L2 (12). With the rising incidence and burden of EAC, tislelizumab utilization is increasing due to its relatively lower economic burden. However, as its application becomes more widespread, reports of related AEs are accumulating.

Endocrine irAEs are among the most common AEs associated with ICIs (8), with an incidence up to 40% (13). Thyroid disorders constitute one of the most frequent endocrine irAEs. A systematic review and meta-analysis estimated the overall incidence of hypothyroidism at 6.6% (14). Among PD-1 inhibitor-related thyroid dysfunction events, tislelizumab exhibited the highest reported incidence at 3.48% (15). Notably, compared to CTLA-4 and PD-L1 inhibitors, PD-1 inhibitors are associated with a higher incidence of thyroid disorders. The reason for this difference remains unclear but may involve the role of PD-L2 blockade in the pathogenesis of thyroid dysfunction, warranting further investigation.

In this case, the patient exhibited normal TSH and fT4 levels prior to tislelizumab initiation. His anti-thyroglobulin antibodies (12.2 IU/mL) and anti-thyroid peroxidase antibodies (12.5 IU/mL) were negative, effectively ruling out classic Hashimoto’s thyroiditis. We systematically excluded other potential etiologies: The patient had no clinical history suggestive of significant iodine deficiency or excess exposure. He was not taking any medications known to induce hypothyroidism (e.g., lithium, amiodarone). Clinical evaluation revealed no signs or historical evidence indicative of pituitary dysfunction. Furthermore, there was no documented recent exposure to iodinated contrast agents preceding the hypothyroidism diagnosis.

The incidence of ICI-DKA is less than 1%. It is a rare but potentially life-threatening complication characterized by acute hyperglycemia, absolute insulin deficiency (serum C-peptide below normal range), ketosis, and acidosis (16). DKA is definitively diagnosed when three criteria are met: (1) Glucose ≥11.1 mmol/1 (200 mg/dl) OR prior history of diabetes; (2)B-Hydroxybutyrate concentration ≥3.0 mmo/OR urine ketone strip 2+ or greater; (3)pH <7.3 and/or bicarbonate concentration <18 mmol/l (17).Endocrine irAEs can manifest at any time after ICI initiation, with symptoms typically appearing within 6 months. However, onset timing is highly unpredictable and can occur at any point during treatment or even months after discontinuation (18). In this case, DKA was diagnosed 11 months after treatment cessation. Therefore, continuous monitoring during ICI therapy and sustained follow-up after discontinuation are crucial.

Diabetic patients are prone to DKA when insulin levels are insufficient. In this case, tislelizumab treatment led to progressive islet dysfunction culminating in severe failure. The potential mechanism for tislelizumab-induced islet destruction involves disruption of immune tolerance: Under physiological conditions, pancreatic β-cells utilize the PD-1/PD-L1 pathway to induce T-cell apoptosis, thereby protecting themselves from cytotoxic immune attack. ICI therapy, while disrupting the PD-1/PD-L1 interaction between tumor cells and T cells, concurrently interferes with the binding of PD-L1 on β-cells to PD-1 on T cells. This leads to β-cell destruction. Studies in nonobese diabetic (NOD) mice indicate that blockade of the PD-1/PD-L1 pathway may increase infiltration of cytolytic IFN-γ+CD8+ T cells into the islets (19, 20). Additionally, genetic predisposition plays a significant role. In Asian populations, human leukocyte antigen (HLA) haplotypes associated with classic T1DM (DR3-DQ2 and DR4-DQ8) and fulminant T1DM (DR4-DQ4 and DR9-DQ9) are overrepresented in ICI-DM patients (21). This suggests a distinct pathophysiology for ICI-induced islet destruction compared to classic T1DM, necessitating further research for better mechanistic understanding.

Previous studies indicate that patients with a history of DM are more susceptible to ICI-induced β-cell destruction (22). Compared to classic T1DM, patients developing ICI-induced islet failure face a higher risk of DKA and a more rapid disease progression, highlighting the dangerous and potentially fatal nature of ICI-DKA (11). Management primarily involves controlling blood glucose, eliminating ketones, and correcting acidosis, specifically with insulin therapy and fluid resuscitation. Lifelong insulin replacement therapy is required subsequently. Glycemic control in ICI-induced diabetic patients poses greater challenges than in classic T1DM patients.

The patient’s pre-ICI antidiabetic regimen (metformin and glimepiride) warrants consideration. Sulfonylureas like glimepiride require residual β-cell function to stimulate insulin secretion. While effective during active tislelizumab therapy (as evidenced by stable FPG), their efficacy diminishes with progressive β-cell loss. The abrupt failure of oral agents observed here reflects the culmination of severe β-cell destruction by tislelizumab, not drug toxicity. The risk stems from the irreversible insulin deficiency induced by ICI-mediated autoimmunity. Nevertheless, clinicians should recognize that non-insulin therapies may mask early β-cell dysfunction in ICI recipients, potentially delaying insulin initiation until DKA occurs.

## 5 Limitations

Our study has limitations. First, due to limited conditions, we confirmed severe islet failure through fasting C-peptide and insulin testing but did not perform HLA genotyping relevant to ICI-DM. Such genetic testing would provide deeper insight into the patient’s condition and be valuable for future research. Furthermore, reporting only a single case inherently limits the generalizability and statistical significance of the findings. Future multi-center registries should prioritize: (1) prospective HLA screening in ICI recipients with diabetes history; (2) longitudinal C-peptide decline tracking to establish predictive biomarkers for βormar failure.

## 6 Conclusion

This case represents the first report of tislelizumab-induced DKA and hypothyroidism in a patient with EAC. The delayed onset of these events posed greater challenges to patient management, highlighting the need for long-term monitoring in patients receiving ICI therapy. ICI therapy can cause delayed destruction of pancreatic β-cells, potentially leading to rapid progression to absolute insulin deficiency and DKA, even in patients with pre-existing diabetes. Monitoring must extend beyond the treatment period since endocrine toxicity can manifest months after discontinuation. We propose mandatory quarterly assessments of fasting glucose, C-peptide, and thyroid function for ≥12 months post-tislelizumab cessation. With the increasing use of tislelizumab for EAC treatment, clinicians should maintain vigilance for its potentially fatal endocrine adverse effects, strengthen monitoring of endocrine function and patient education, and implement timely interventions to prevent DKA.

## Data Availability

The original contributions presented in the study are included in the article/**Supplementary Material**. Further inquiries can be directed to the corresponding authors.
